# The effect of heat therapy on pain intensity, duration of labor during first stage among primiparous women and Apgar scores: A systematic review and meta-analysis

**DOI:** 10.18332/ejm/156487

**Published:** 2022-11-28

**Authors:** Sujata Goswami, Prasuna Jelly, Suresh K. Sharma, Rizu Negi, Rakesh Sharma

**Affiliations:** 1 College of Nursing, All India Institute of Medical Sciences, Rishikesh, India; 2 College of Nursing, All India Institute of Medical Sciences, Jodhpur, India

**Keywords:** hot temperature, warm compress, labor, pain, Apgar score, pain management, uterine contraction, obstetric

## Abstract

**INTRODUCTION:**

Heat therapy may help in reducing pain during labor as it blocks the receptors of pain, according to gate control theory. This systematic review and meta-analysis study aims to evaluate the effect of heat therapy (HT) systematically and critically on pain intensity, duration of labor during the first stage of labor and Apgar scores.

**METHODS:**

We searched for randomized controlled trials published until October 2020 in PubMed/Medline, EMBASE, ClinicalKey, Ovid Discovery, and other sources. Randomized controlled trials (RCTs) comparing heat therapy with standard treatment were selected.

**RESULTS:**

Out of 7625 screened, 10 studies met the inclusion criteria. The results of pooled data have shown that heat therapy was significantly effective in reducing pain intensity in the first stage of labor (standard mean difference, SMD= -1.31; 95% CI: -1.88 – -0.73; p<0.001). Heat therapy had significantly reduced the duration of the first stage of labor (pooled MD= -50.09; 95% CI: -89.70–10.48; p=0.01) and was also superior to the standard therapy group in terms of better Apgar scores at the 5th minute of birth of the newborn (pooled MD= -0.10; 95% CI: -0.19–0.02; p=0.02).

**CONCLUSIONS:**

Current evidence shows that heat therapy effectively decreases labor pain intensity and shortens the duration of labor in the first stage, and it can be used as nonpharmacological management for labor pain.

## INTRODUCTION

Pain during labor has been described as one of the most severe pains experienced by most women in their life^1,2^. Labor is a normal event in a mother’s life^3^. Consistently, painful labor contractions occur with a decrease in cervical dilation and/or effacement^4^. Mechanical stretch and hormones can work together to start contractions in normal labor, although the exact causes of uterine contractions are not known^5,6^. Stretch (distension) is a contractile stimulus to smooth muscle. Estrogen, progesterone, prostaglandins, and oxytocin are the main hormones involved in uterine contractions^7,8^.

Globally, there are several non-pharmacological (hypnosis, biofeedback, intracutaneous or subcutaneous sterile water injection, immersion in water, aromatherapy, relaxation techniques, yoga, music, audio, massage, etc.) and pharmacological measures (inhaled analgesia, opioids, non-opioid drugs, epidural including combined spinal-epidural, local anesthetic nerve blocks) are used for the management of pain during labor^9-14^.

However, pharmacological methods have several hazardous side effects for the mother and baby (e.g. decreased cardiac output, nerve damage, allergy, and diminished progression of the second stage of labor)^15-18^. Non-pharmacological measures are reported to be more safe for the mother as well as the baby^11^, and many nurses and midwives shared their experiences and reported that most of them were using these measures while caring for mothers during the childbirth process to manage labor pain^19^.

Local heat therapy application causes blood vessels to dilate, improving blood supply and momentarily stopping pain signals from reaching the brain^20^. The cutaneous and deeper tissues appear to be stimulated by heat. According to gate control theory, thermoreceptors can help to reduce pain^21^. Thermotherapy is a modern non-pharmacological tool for inducing uterine contractions and shortening the labor period^22^. The influence of thermotherapy on labor pain was favored by 80.4% of midwives and 79.7% of birthing women. The majority of midwives recommend continuing to use heat therapy for other participants^21^.

Most clinical trials favored heat therapy as one of the most apposite, safe, and cost-effective interventions in the management of pain during the first stage of labor. Although, a recent Cochrane review by Smith et al.^23^ reported that there was a significant reduction in pain during the first stage of labor among mothers who received heat therapy than routine treatment. In the review, they included randomized controlled trials (RCTs), quasi-RCTs, even cluster studies, even available abstracts only, and trials with sequential intervention^20^ of cold and hot applications. Furthermore, few trials were missed^24,25^, and few got published after this Cochrane review publication^26-28^. Thus, this systematic review (SR) and meta-analysis (MA) study aimed, keeping in mind sound methodological planning, to provide strong evidence regarding the efficacy of heat therapy. Therefore, the objective of this study was to systematically examine RCTs on the effect of heat therapy on labor pain intensity, uterine contractions, labor duration during the first stage of labor, and Apgar scores at the 1st and 5th minute with a well-framed research question: ‘Does heat therapy affect pain intensity, uterine contractions, and duration of labor phases during the first stage of labor in a primigravida mother compared to a mother receiving routine care’.

## METHODS

### Aim

The current review aims to evaluate the effectiveness of heat therapy to reduce labor pain intensity, uterine contractions, duration of labor during the first stage, and Apgar scores at the first and fifth minute of birth.

### Design

**Figure 1:**
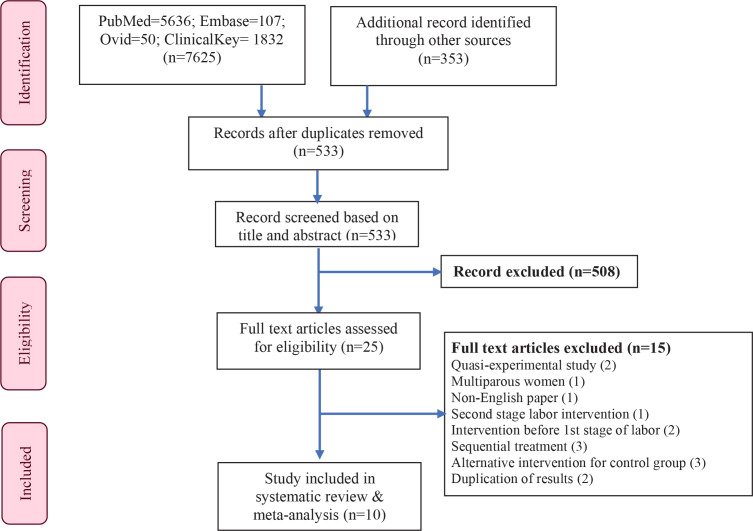
PRISMA flow diagram

We followed the Preferred Reporting Items for Systematic Review and Meta-Analysis (PRISMA)^29^ (Figure 1) guidelines for this systematic review and meta-analysis. The PICO (patient/population, intervention, comparison, and outcomes) framework was used to justify the review question. The review was registered at the International Prospective Register of Systematic Reviews (PROSPERO) (https://www.crd.york.ac.uk/PROSPERO/, registration number: CRD42020219730).

### Search method

We used PubMed/Medline, Embase, Ovid, and ClinicalKey as major electronic databases to search literature from the time of inception to September 2021, in the English language. Studies were searched that assessed the effect of heat therapy on pain intensity, uterine contractions, and duration of labor during the first stage of labor among primiparous women along with the effect of heat therapy on Apgar scoring at the first and fifth minute.

The MeSH (medical subject headings) and search terms related to: [hot temperature OR thermoregulation OR hot OR steam OR warm] AND [labor OR obstetric pain]; [hot temperature OR thermoregulation OR hot OR steam OR warm] AND [labor OR labor onset OR labor duration 1st stage]; [hot temperature OR thermoregulation OR hot OR steam OR warm] AND [uterine contractions OR myocardial activity]; [hot temperature OR thermoregulation OR hot OR steam OR warm] AND [Apgar score]. Boolean operators (AND and OR) and truncation were used in the search. The search strategy details are given in the Supplementary file Table 1. We examined the reference lists of articles to identify additional studies and searched the grey literature with Google.

**Table 1:** Characteristics of included studies comparing heat therapy (HT) with standard treatment (ST)

*Author Year Country*	*Participant characteristics*		*Sample size (HT/ST)*	*Heat therapy*		*Outcome*			
	*Age (years) Mean ± SD or Range*	*GA (weeks) Mean ± SD or Range*		*Type and site*	*Temperature and duration (minutes)*	*PI (1st stage) Mean ± SD*	*Labor duration (minutes) Mean ± SD*	*AS at 1 minute Mean ± SD*	*AS at 5 minute Mean ± SD*
Kaur et al.^26^ 2020 India	18–35		44/44	Warm compress, lumbo-sacral area	70℃ 3 times with a gap of 1 hour	HT=7.55 ± 0.87 ST=7.68 ± 0.97			
Tarrats et al.^27^ 2019 Spain	31 ± 6	39 ± 3	67/67	Thermal belt with 2 pockets, lumbar and suprapubic area	38–39℃ 30 min	HT=6.90 ± 0.74 ST=5.50 ± 0.71		HT=8.66 ± 1.27 ST=8.98 ± 0.48	HT=9.75 ± 0.79 ST=9.98 ± 0.12
Farahmand et al.^28^ 2019 Iran	18–35	37–42	75/75	Warm compress, perineum	70℃ 15–20 min	HT=5.35 ± 1.59 ST=8.61 ± 1.05			
Akbarzadeh et al.^22^ 2018 Iran	18–35	37–42	74/75	Warm compress, perineum	70℃ 15–20 min		HT=169.89 ± 34.74 ST=196.58 ± 42.53 (p=0.267)	HT=8.95 ± 0.19 ST=8.97 ± 0.16	HT=9.9 ± 0.11 ST=9.89 ± 0.115
Eckert et al.^24^ 2001 Australia	27 ± 6	≥37	137/137	Hot bath, full body	38℃		HT=404.23 ± 225.23 ST=407.21 ± 222.56 (p=0.89)	HT=9.00 ± 1.7 ST=9.00 ± 1.5	HT=9.00 ± 0.7 ST=9.00 ± 0.6
Behmanesh et al.^34^ 2009 Iran	18–35	37–41	32/32	Hot water bag, lower back and perineum	80 min	HT=8.14 ± 0 .99 ST=8.88 ± 1.20	HT=161.56 ± 73.97 ST=219.84 ± 50.63		
Yazdkhasti et al.^25^ 2018 Iran	18–35	>37	35/34	Hot water bottle, lower back and abdomen	60 min (compared with ST of 10 min in every 30 min)	HT=6.00 ± 1.35 ST=7.80 ± 1.18	HT=293.70 ± 68.97 ST=400.86 ± 77.43 (p=0.001)	HT=8.68 ± 0.47 ST=8.68 ± 0.47	HT=9.85 ± 0.35 ST=9.97 ± 0.16
Taavoni et al.^30^ 2013 Iran	18–35	38–40	30/30	Warm pack via moist towel, sacrum and perineum	45℃ 30 min	HT=8.08 ± 1.47 ST=9.29 ± 1.10			
Lee et al.^35^ 2013 Taiwan	31–64		39/41	Warm shower, full body and lower back	37℃ 20 min	HT=7.10 ± 1.92 ST=8.85 ± 1.22			
Silva et al.^36^ 2007 Brazil	15–31	37–41	54/54	Immersion bath	<38℃ 60 min	HT=8.5 ± 1.6 ST=9.3 ± 1.4		HT=8.7 ± 0.5 ST=8.8 ± 0.5	HT=9.4 ± 0.5 ST=9.5 ± 0.5

AS: Apgar score. GA: gestational age. PI: pain intensity. p<0.05 level of significance.

### Study selection criteria

The references were imported into Mendeley (Desktop version 1.19.8), a reference management software program. All the studies were screened and only randomized controlled trials were included.

*Inclusion criteria*

The inclusion criteria were: 1) randomized controlled or controlled clinical trial on humans; 2) mothers aged 18–40 years who were in the first stage of the labor process, admitted in a labor room for normal vaginal delivery; 3) normal primipara mothers with 37–40 weeks period of gestation, with single live fetus in cephalic presentation; 4) studies comparing heat therapy versus standard treatment for pain management, Apgar scores, and uterine contractions; and 5) full-text articles in the English language.

*Exclusion criteria*

The exclusion criteria were: single live fetus in the cephalic presentation in an abnormal position, i.e. posterior, and transverse position.

According to predetermined eligible criteria, two authors (SG, RN) screened the titles and abstracts of all the articles. PJ and RS finally judged all the disagreements related to the inclusion and exclusion criteria of studies. A thorough review of full-text articles was done by SG and RN. A final decision on the inclusion of studies was made after a discussion with the review team members.

### Types of intervention and control

We included all the trials examining the effect of heat therapy on any area like abdomen, perineum, suprapubic area, lumbar and sacral area for pain relief, uterine contractions, and labor duration of the first stage of labor. We excluded all other invasive or non-invasive methods for relieving pain like analgesic drugs, anesthesia, acupressure, massage, and combined therapies. Standard care or no treatment without any form of pain relief was considered as the comparison group. Heat therapy was applied by different methods like warm compress via moist towel, hot bath, immersion, hot water bag, and thermal belt with two pockets.

### Outcome measures and data extraction

All authors gathered a predefined outcome from studies, which includes study characteristics. The primary outcomes for this review were pain intensity, Apgar scores at the first and fifth minute of birth, and duration of labor during the first stage. Secondary outcomes were uterine contractions.

Data extraction was carried out by excluding duplicate studies. Two independent reviewers (SG, RN) extracted data in a data extraction sheet, and two reviewers (PJ and RS) cross-checked all the data. The formal discussions and consensus by the senior reviewers (RS and SKS) resolved the differences. The corresponding author was contacted in case any additional information required. We attempted to contact nine study corresponding authors, but only two authors^27,30^ responded to our emails.

### Quality assessment

The methodological quality of the ten studies was assessed using the Cochrane Collaboration approach to assess risk bias, shown in Supplementary file Figures 1 and 2. In a disagreement between authors, RS and SKS were consulted to reach a final decision. All the studies were assessed based on six criteria: random sequence generation, allocation concealment (selection bias), blinding of participants and personnel (performance bias), blinding of outcome assessor (detection bias), incomplete outcome data (attrition bias), selective outcome reporting (reporting bias), and other biases (contamination between experimental and control group).

All the included studies were classified as low risk, high risk, or unclear risk. If a study reported a low risk for all domains of risk bias, it was considered high quality and vice versa. If there was a difference of opinion between the primary reviewers (SG, PJ, RN) regarding risk of bias, senior reviewers (RS, SKS) conducted a thorough assessment of the studies, and conclusions were reached with mutual consensus. A subjective report on the risk of bias is given in Supplementary file Table 2.

### Statistical analysis

The meta-analysis was done by using Review Manager Software (RevMan version 5.4.1)^31^.

Mean and standard deviation (SD) scores for each study were entered into the software to draw forest plots of various outcomes. Outcomes like pain intensity and labor duration during the first stage were continuous data and represented as standardized mean difference (SMD) with 95% confidence intervals. Heterogeneity was tested both by visual examination of a forest plot (where non-overlapping confidence interval (CI) shows the probability of heterogeneity) and by use of chi-squared heterogeneous test. Heterogeneity was represented as I^2^ (%) with: 0% no heterogeneity, 25% low heterogeneity, 50% moderate heterogeneity, and 75% high heterogeneity^32^. A weighted inverse-variance random-effects model was considered to compare between the groups. We used a reference value of I^2^ >75% to indicate substantial variability related to heterogeneity^32,33^. To identify the publication bias, a funnel plot was drawn and assessed by visual inspection for its symmetry (Supplementary file Figure 3).

## RESULTS

Details of the study selection process and search results are presented in Figure 1. A total of 7625 articles were found corresponding to the search strategy and based on the systematic literature search of four major databases: PubMed (n=5636), EMBASE (n=107), Ovid (n=50) and ClinicalKey (n=1832).

Additional searches from other sources such as manual searches through reference lists of articles and search engines like Google Scholar were attempted. A total of 353 articles were retrieved from Google scholar. No new articles were found from manual tracking.

After duplication removal (7445), the title and abstract of 533 articles were screened. Twenty-five studies were eligible for full-text review. After reviewing, fifteen articles were excluded due to various reasons (quasi-experimental =2; multipara women as participants =1; published in Persian language =1; intervention was in the second stage =1; administered intervention before the first stage of labor =2; sequential treatment =3; duplication of results =2 and alternative treatment in the control group =3). Finally, 10 studies were included which are summarized in Table 1 and Figure 1.

### Characteristics of included studies and patients

Ten studies were included in this systematic review and meta-analysis, with 1185 participants, 587 in the experimental and 598 in the control group. The studies were published between the years 2001 and 2020. The sample sizes of the studies ranged from 28 to 137. Out of 10 included studies, five^22,25,28,30,34^ were performed in Iran, and the remaining five were from Taiwan^35^, Brazil^36^, India^26^, Australia^24^, and Spain^27^. The age of the participants ranged from 18 to 35 years (Table 1).

### Details of heat therapy and control types

Heat therapy as an intervention was given to mothers by different methods in selected studies by obstetricians and midwives. These methods were warm pack^22,26,28^, hot water bag^30,34^, warm shower^35^, moist towel^30^, thermal belt with two pockets^27^, hot bath^24^, and immersion in warm water^36^. The duration of heat therapy was different with different temperatures (70°C for 15 min, and 38°C for 60 min).

Among all the studies, pain intensity and duration of labor during the first stage were assessed as outcomes. In most studies, pain was measured using a visual analogue scale^22,27,30,35^, and a numeric rating scale^26,34,36^; whereas, the McGill pain questionnaire^25^ was used in only one study. Digital watch and partograph were used for assessing duration of the first stage of labor. Secondary outcomes of this study were Apgar scores at first and fifth minute of birth, which were assessed in five studies^22,25,27,34,36^.

### Methodological quality of the studies

Random sequence generation was described in nine studies^22,25-28,30,35-37^, and adequate concealment was reported in four studies^24,25,27,36^. Blinding of personnel and participants was mentioned in one study^36^, and no sufficient information was provided in the study by Eckert et al.^24^, so it has an unclear risk of bias. In only two trials^26,30^ outcome assessors were blinded, and one study^24^ not explained. The majority of the included RCTs were considered low risk for incomplete outcome data bias; only one study^22^ had an unclear risk of bias. Based on our judgement, except for one study^24^, all had a low risk of selective reporting bias. In other risks of bias, three studies^22,26,28^ were at low risk of bias, and the remaining had a high risk of bias. In the incident of any missing information from the study findings, all authors were consulted/informed, and after receiving responses from the corresponding authors of the included studies, further decisions were made with the mutual consensus of all authors of this analysis.

### Primary outcomes

*Pain intensity during the first stage of labor*

**Figure 2:**
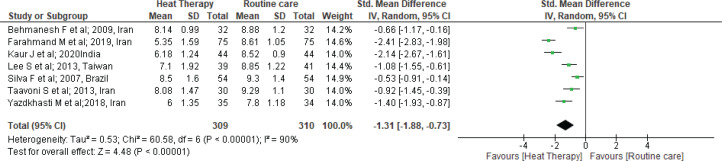
Forest plot of comparison: Heat therapy versus standard treatment; Outcome: Pain intensity in 1st stage

Seven studies^25,26,28,30,34-36^ were identified, as shown in Figure 2, which evaluated the effect of heat therapy on pain reduction at the first stage among 619 primiparous women (heat therapy, HT=309; standard therapy, ST=310). Pooled results from the studies by random effect model demonstrated that there was a significant reduction in the pain intensity among the heat therapy group during the first stage of labor in comparison to standard treatment (SMD= -1.31; 95% CI: -1.88 – -0.73; I^2^=90%; p<0.001) (Figure 2).

**Figure 3:**
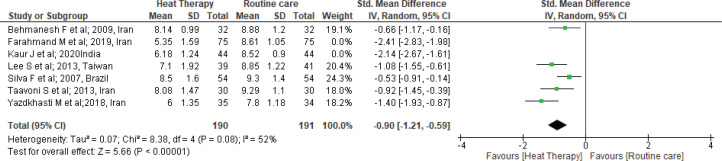
Forest plot of comparison: Heat therapy versus standard treatment; Outcome: Pain intensity in 1st stage

Furthermore, we performed sensitivity analysis. One by one, the studies were checked and two studies^26,28^ that had a high level of heterogeneity were removed (SMD= -0.90; 95% CI: -1.21 – -0.59; I^2^=52%; p<0.001) (Figure 3).

*Duration of first stage of labor*

**Figure 4:**
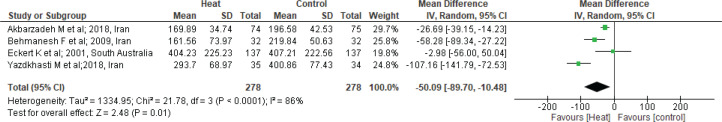
Forest plot of comparison: Heat therapy versus standard treatment; Outcome: Labor duration in 1st stage

The duration of the first stage of labor was measured by four trials^22,24,25,34^ comprising 306 mothers in HT and 307 in ST groups, as shown in Figure 4. Pooled effect size from the random effect model shows that there was no significant difference in the duration of the first stage of labor between HT and ST groups (MD= -35.49; 95% CI: -72.45–1.47; I^2^= 90%; p=0.06).

**Figure 5:**
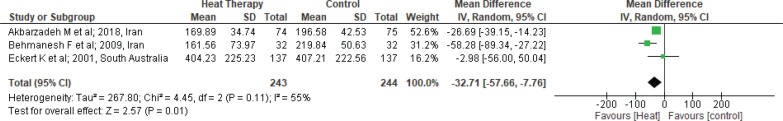
Forest plot of comparison: Heat therapy versus standard treatment; Outcome: Labor duration in 1st stage

There was great variability observed among the four studies, which caused heterogeneity in the results. In two studies^25,34^, a hot water bag was used for heat therapy. Additionally, the site for heat application was different in all the studies. The duration of HT was different in all the studies, ranging from 15 min to 80 min. To find the reason for the heterogeneity, a sensitivity analysis was performed; after excluding one study^25^ as it was causing high heterogeneity. Further analysis found significant difference in the duration of first stage of labor between HT and ST groups, as shown in Figure 5, with 55% heterogeneity (MD= -32.71; 95% CI: -57.66 – -7.76; I^2^= 55%; p=0.01).

*Apgar score in the first minute*

The effect of heat therapy on Apgar score in the first minute of birth was measured in five trials^22,24,25,27,36^, including 734 mothers (367 in each group). A forest plot was drawn (Supplementary file Figure 4) showing no significant effects of heat therapy on Apgar score of babies in the first minute of birth between the HT group and ST group, from the pooled results with the fixed-effect model (MD= -0.03; 95% CI: -0.08–0.02, I^2^ =0%; p=0.22).

*Apgar score in the fifth minute*

A forest plot given in Supplementary file Figure 5 shows the analysis of five studies^22,24,25,27,36^, measuring the effects of heat therapy on Apgar score of babies in the fifth minute of birth. The number of mothers was equal in both groups (367 in each).

After pooled proportion of the result by a random effect model, it was reported that MD was -0.07 (95% CI: -0.15–0.02; I^2^= 60%; p=0.14), which was not statistically significant. To understand which study caused heterogeneity, we removed a trial^22^, and we found that there was a significant difference between the heat and control groups regarding the Apgar score enhancement after the birth of a baby, as shown in Supplementary file Figure 6 (MD= -0.10; 95% CI: -0.19 – -0.02; I^2^ =14%; p=0.02).

### Secondary outcome

*Uterine contractions*

Authors were unable to perform meta-analysis of this outcome, as none of the studies measured the effects of heat therapy on uterine contractions during the first stage of labor among primipara mothers.

### Publication bias

The publication bias for Apgar score at the 5th minute chosen outcome was evaluated using a funnel plot in Supplementary file Figure 3, which revealed a symmetrical pattern, suggesting that there was no publication bias.

## DISCUSSION

This review aimed to investigate the effectiveness of heat therapy in relieving labor pain, uterine contraction, labor duration during the first stage of labor, and Apgar scores at the 1st and 5th minute. Midwives often recommend local heat application as a pain relief method during labor. Therefore, the efficacy of heat therapy is given by different methods, including elastic belt, moist towel, warm packs, warm tub, and hot water bottle. There were various sites the heat therapy was applied: on the lumbosacral, abdomen, perineum, supra-pubic area, and lower back, which was evaluated in this systematic review and meta-analysis. In this regard, the Visual Analog Scale, Numerical Pain Rating Scale, and McGill Questionnaire were used to quantify the intensity of maternal pain as a valid, reliable, and subjective pain assessment instrument and partograph for the duration of the first stage of labor. Furthermore, randomized controlled trial with a suitable control group accurately reflected the effects of this treatment.

This study’s findings suggest that heat therapy has an additive role in reducing pain during the first stage of labor. Heat therapy blocks the transmission of impulses to the brain by releasing endorphins to decrease pain according to gate control theory^21^. Moreover, it increases the secretion of oxytocin, decreases the production of adrenalin, and eventually results in labor progress, which ultimately leads to reduced labor duration^22^. This review showed a significant difference in favor of heat therapy in the first stage of labor.

According to the present review, we may claim that the application of heat therapy either in the form of dry heat (e.g. hot water bag, warm packs) or in the form of moist heat (e.g. immersion, warm shower, warm bath) are beneficial in reducing pain for the laboring mothers. Similarly, a study^38^ has shown that women who received moist heat (warm bath) during the first stage of labor had reduced labor pain compared to women who received routine care. In addition, literature^26,30^ has reported that moist heat therapy tends to stimulate deeper tissues; therefore, it is more beneficial during labor for pain reduction.

The application site of heat therapy also plays a significant role in reducing labor pain during the first stage. In the present study, five trials^25,26,30,34,35^ applied heat therapy to the sacral and lower back areas during the first stage of labor, which helped in reducing pain.

A similar mechanism was observed where massage therapy was given that helped in improving blood flow, which resulted in relieving pain and exhaustion among mothers during labor^39,40^. In massage therapy, heat is generated that reduces sensitivity, muscle stiffness and blocks the transmission of impulses to the brain via the release of endorphins^41^. Local heat application increases the elasticity of the collagen tissues, which helps increase tissue flexibility and reduce the severity of pain^42^.

In the present review, the effect of heat therapy on the duration of labor during the first stage was significantly reduced with the pooled data of three trials^22,24,34^. Similar findings were reported, where researchers used warm bags^34^ and hot water bags^25^ over the lower back and abdomen during labor, which helped in reducing the duration of labor of mothers in the intervention group and then the routine care group. In other studies, mothers were given a warm shower during labor, which decreased the duration of labor, not only in the first stage but also in the second stage^30,43^. In contrast, heat therapy given by warm towel^37^, warm packs^22^, and by immersion in a warm bath^24^, was not effective in reducing the duration of the first stage of labor.

We did not perform meta-analysis, as there was no trial on the effect of heat therapy on uterine contraction during the first stage of labor. A study^44^ reported that heat therapy significantly increased uterine contractility in multiparous mothers during the labor process.

Adverse outcomes of a neonate, such as low Apgar score and increased admission to neonatal intensive care units, were significantly associated with increased duration of labor^44^. Similarly, in the present study, heat therapy has shown no impact on the neonatal Apgar score at the 1st minute but significantly increased at the 5th minute in the heat therapy group.

It is important to study the adverse impacts of any intervention. In a study, maternal and neonatal effects (fetal heart rate, fetal distress, and referral to intensive care) of a hot bath were measured, but no such outcomes were reported^21^.

Another study reported that after heat therapy there was a rise in fetal heart rate and all the newborns were healthy, with no harmful effects in newborns and mothers reported^45^. In addition to the reduction of pain, duration of labor, and higher Apgar score at the 5th minute, there were other benefits of heat therapy, including physical hygiene effects, more comfort, and more satisfaction reported by mothers who received heat therapy by warm shower than those who got routine care during labor^35^.

### Limitations

Our study has some limitations. Firstly, we only included articles published in English; as a result, it was possible that some of the significant trials could have been missed from the outcomes of the present synthesis. Secondly, studies included in the present meta-analysis were heterogenous and, therefore, findings of the study need to be utilized carefully in clinical practice.

## CONCLUSIONS

Heat therapy was found to be effective in pain management during the first stage of labor and shortened the birthing duration. Overall, the data show that heat has an additive advantage when used in place of standard treatment. Heat therapy can be used as a non-pharmacological measure to manage pain during the first stage of labor. Although Apgar score was not effective at the first minute after birth, it was significantly effective at the 5th minute. There is a great need for more high-quality trials, as most of the included studies in this review were found to have a high risk of bias. Moreover, there is a need for more RCTs on the effects of heat therapy on uterine contractions.

## Data Availability

The data supporting this research can be found in the Supplementary file.
