# Supplementary material for: The effect of heat therapy on pain intensity, duration of labor during first stage among primiparous women and Apgar scores: A systematic review and meta-analysis
Source: Eur J Midwifery. 2022 Nov 28;6:66. doi: 10.18332/ejm/156487 (PMC9703937; doi:10.18332/ejm/156487)
Supplement: Supplementary file 1 [file EJM-6-66-s1.pdf]

Supplementary file-1: Search Strategy Details

PubMed Central Search Strategy

("hot temperature/therapeutic use"[MeSH Terms] OR "hot temperature/therapy"[MeSH Terms]) AND ("labor s"[All Fields] OR "labored"[All Fields] OR "laborer"[All Fields] OR "laborer s"[All Fields] OR "laborers"[All Fields] OR "laboring"[All Fields] OR "labors"[All Fields] OR "labour"[All Fields] OR "work"[MeSH Terms] OR "work"[All Fields] OR "labor"[All Fields] OR "labor, obstetric"[MeSH Terms] OR ("labor"[All Fields] AND "obstetric"[All Fields]) OR "obstetric labor"[All Fields] OR "laboured"[All Fields] OR "labourer"[All Fields] OR "labourers"[All Fields] OR "labouring"[All Fields] OR "labours"[All Fields])

("hot temperature/therapeutic use"[MeSH Terms] OR "hot temperature/therapy"[MeSH Terms]) AND "Uterine Contraction"[MeSH Terms]

"hyperthermia, induced"[MeSH Terms] AND "Apgar Score"[MeSH Terms]

"Steam Bath"[MeSH Terms] AND ("labor, obstetric"[MeSH Terms] AND "labor stage, first"[MeSH Terms])

"Steam Bath"[MeSH Terms] AND ("labor pain"[MeSH Terms] OR ("labor"[All Fields] AND "pain"[All Fields]) OR "labor pain"[All Fields])

("hydrotherapy/therapeutic use"[MeSH Terms] OR "hydrotherapy/therapy"[MeSH Terms]) AND ("Apgar Score"[MeSH Terms] AND 2019/01/01:2020/12/31[Date - Publication])

"warm"[All Fields] AND ("labor pain"[MeSH Terms] OR ("labor"[All Fields] AND "pain"[All Fields]) OR "labor pain"[All Fields])

("immerse"[All Fields] OR "immersed"[All Fields] OR "immerses"[All Fields] OR "immersing"[All Fields] OR "immersion"[MeSH Terms] OR "immersion"[All Fields] OR "immersions"[All Fields] OR "immersive"[All Fields] OR "immersiveness"[All Fields]) AND "Uterine Contraction"[MeSH Terms]

"warm"[All Fields] AND ("labor s"[All Fields] OR "labored"[All Fields] OR "laborer"[All Fields] OR "laborer s"[All Fields] OR "laborers"[All Fields] OR "laboring"[All Fields] OR "labors"[All Fields] OR "labour"[All Fields] OR "work"[MeSH Terms] OR "work"[All Fields] OR "labor"[All Fields] OR "labor, obstetric"[MeSH Terms] OR ("labor"[All Fields] AND "obstetric"[All Fields]) OR "obstetric labor"[All Fields] OR "laboured"[All Fields] OR "labourer"[All Fields] OR "labourers"[All Fields] OR "labouring"[All Fields] OR "labours"[All Fields])

Embase

('heat'/exp OR heat) AND ('labor pain'/exp OR 'labor pain')

(('heat'/exp OR heat) AND ('uterus contraction'/exp OR 'uterus contraction')) AND 'randomized controlled trial'/de

('thermotherapy'/exp OR thermotherapy) AND ('uterine cervix ripening'/exp OR 'uterine cervix ripening')

('thermotherapy'/exp OR thermotherapy) AND ('delivery stage 1' OR (('delivery'/exp OR delivery) AND stage AND 1))

(('thermoregulation'/exp OR thermoregulation) AND ('labor pain'/exp OR 'labor pain')) AND 'randomized controlled trial'/de

('thermoregulation'/exp OR thermoregulation) AND ('delivery stage 1' OR (('delivery'/exp OR delivery) AND stage AND 1))<br>('thermal conductivity'/exp OR 'thermal conductivity') AND ('uterus contraction'/exp OR 'uterus contraction')<br>('thermal exposure'/exp OR 'thermal exposure') AND ('apgar score'/exp OR 'apgar score')<br>('heat stress'/exp OR 'heat stress') AND ('apgar score'/exp OR 'apgar score')<br>('heat tolerance'/exp OR 'heat tolerance') AND ('uterus contraction'/exp OR 'uterus contraction')<br>('heat transfer'/exp OR 'heat transfer') AND ('uterine cervix ripening'/exp OR 'uterine cervix ripening')<br>('water vapor'/exp OR 'water vapor') AND ('delivery stage 1' OR (('delivery'/exp OR delivery) AND stage AND 1))<br>('water vapor'/exp OR 'water vapor') AND ('labor pain'/exp OR 'labor pain')
**Ovid:**<br>(hot temperature OR thermoregulation OR hot OR steam OR warm AND labor or obstetric pain).mp. [mp=title, abstract, full text, caption text]<br>(hot temperature OR thermoregulation OR hot OR steam OR warm AND labor or Labor onset or labor duration 1st stage).mp. [mp=title, abstract, full text, caption text]<br>(hot temperature OR thermoregulation OR hot OR steam OR warm AND uterine contractions OR myocardial activity) mp. [mp=title, abstract, full text, caption text]<br>(hot temperature OR thermoregulation OR hot OR steam OR warm AND Apgar score) mp. [mp=title, abstract, full text, caption text]
**Clinical key**<br>(hot temperature OR thermoregulation OR hot OR steam OR warm AND labor or obstetric pain).<br>(hot temperature OR thermoregulation OR hot OR steam OR warm AND labor or Labor onset or labor duration 1st stage).<br>(hot temperature OR thermoregulation OR hot OR steam OR warm AND uterine contractions OR myocardial activity)<br>(hot temperature OR thermoregulation OR hot OR steam OR warm AND Apgar score)
**Google & google scholar**<br>(hot temperature OR thermoregulation OR hot OR steam OR warm AND labor or obstetric pain).<br>(hot temperature OR thermoregulation OR hot OR steam OR warm AND labor or Labor onset or labor duration 1st stage).<br>(hot temperature OR thermoregulation OR hot OR steam OR warm AND uterine contractions OR myocardial activity)<br>(hot temperature OR thermoregulation OR hot OR steam OR warm AND Apgar score)

PICO Framework

**Population**	Primiparous women aged between 18-40 years
**Intervention**	Heat therapy
**Comparison**	Standard treatment with routine care and regular treatment

Outcome	**Primary Outcome:** Pain intensity, uterine contractions and duration of labor in first stage.<br>**Secondary Outcome:** Apgar score at 1st and 5th minute

Supplementary file-2: Risk of bias graph: review authors' judgments about each risk of bias item presented as percentages across all included studies.

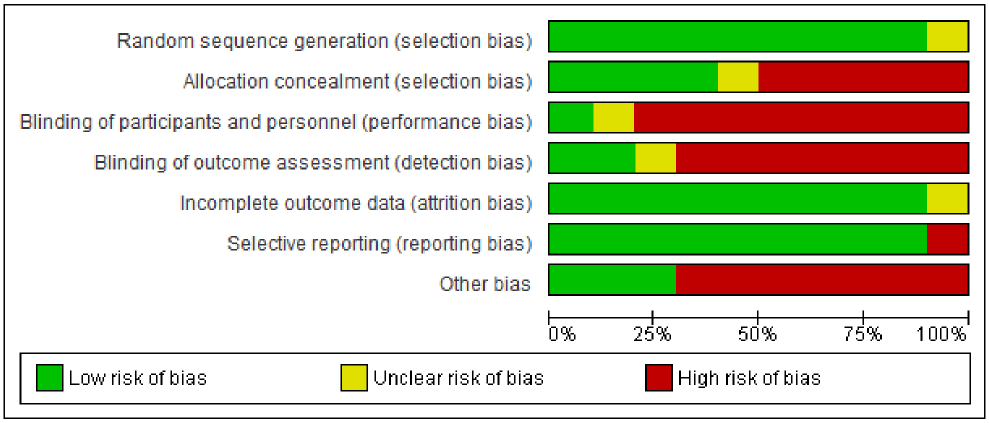

Supplementary file-3: Risk of bias summary: review authors' judgments about each risk of bias item for each included study.

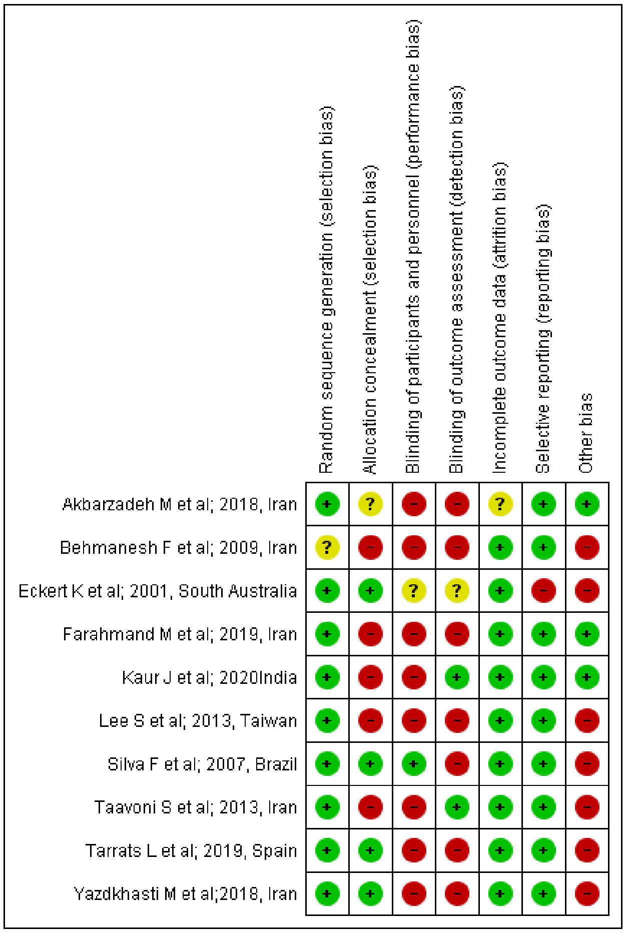

Supplementary file-4: Risk of bias assessment using Cochrane checklist

Study	Randomization (Selection Bias)	Allocation (Selection Bias)	Participant & Personnel Blinding (Performance Bias)	Outcome Blinding (Detection Bias)	Attrition Bias	Selective Reporting	Other Bias
**1. Kaur J et al; 2020 India**	**Low** Randomized with standardized method i.e., **Randomized computer-generated table**	**High** Opaque sealed and sequentially numbered envelops were not used	**High** Participants were not blinded	**Low** Medical assessors were blinded to treatment	**Low** Complete outcome data reporting	**Low** All the study outcomes are reported	**Low** All sufficient information was provided regarding consort and trials registration.
**2. Tarrats L et al; 2019 Spain**	**Low** Randomized with standardized method i.e., **Randomized computer-generated table**	**Low** They used sealed, opaque, sequentially numbered envelops for allocation concealment.	**High** Participants were not blinded	**High** Not blinded.	**Low** They have mentioned the reason for drop outs.	**Low** All the study outcomes are reported	**High** CONSORT diagram was not given.
**3. Farahmand M et al; 2019 Iran**	**Low** All mothers were randomly assigned in blocks of 4.	**High** Opaque sealed and sequentially numbered envelops were not used	**High** Participants were aware of their treatment as they discussed treatment with physician or research team	**High** Outcome assessors were not blinded in this research trial	**Low** Complete outcome data reporting	**Low** All the study outcomes were clearly reported.	**Low** Information was sufficient to provide judgement
**4. Akbarzadeh M et al; 2018 Iran**	**Low** Randomized with standardized method i.e., **Randomization Table**	**Unclear** Allocation was mentioned but there was no mention that how it was done	**High** They have mentioned that, it was not possible to blind the study since both delivery and intervention were conducted in the same environment	**High** There was no mention of outcome assessor blinding	**Unclear** 1 participant lost to follow-up but they have not provided reason	**Low** All the study outcomes were clearly reported	**Low** Information was sufficient.
**5. Eckert K et al; 2001 South Australia**	**Low** Randomized with standardized method i.e., **Randomization Table**	**Low** Clear mention of allocation concealment i.e., sealed opaque envelopes	**Unclear** It was mentioned that conventional additional treatment was given to both the group which is indicative of blinding but not clear	**Unclear** Outcome was assessed by midwives but name of researcher is not there.	**Low** They have mentioned the reason for drop outs.	**High** 31 women with group B streptococcal colonization were not excluded and were entered into the trial	**High** Registration of clinical trial is not given.
**6. Behmanesh F et al; 2009 Iran**	**Unclear** They mentioned about randomly division but not	**High** Not mentioned about whether envelops were opaque or	**High** Participants blinding were not mentioned	**High** Not mentioned about outcome assessor blinding	**Low** Complete outcome data reporting, no missing data	**Low** All the study outcomes were clearly reported	**High** Information about registration trial and CONSORT flow chart is not provided.

	explained how they have done it.	sequentially numbered					
**7. Yazdkhasti M et al;2018 Iran**	**Low** Randomization process was mentioned i.e., **computer-based randomization**	**Low** Clear mention of allocation concealment i.e., sealed opaque envelopes	**High** Blinding of participants not mentioned	**High** Blinding of outcome assessors not mentioned	**Low** Clearly mentioned about participants who withdrew from study	**Low** All the study outcomes were clearly reported	**High** Data about registration trial is not mentioned.
**8. Taavoni S et al; 2013 Iran**	**Low** Randomized with standardized method i.e., **Randomization Table**	**High** Not mentioned	**High** Blinding of participants not mentioned	**Low** Statistician was blind	**Low** They have mentioned the reason for drop outs.	**Low** All the study outcomes were clearly reported	**High** Not mention about CONSORT and registration.
**9. Lee S et al; 2013 Taiwan**	**Low** Randomization process was done by **computer software.**	**High** Not mentioned about sealed or opaque envelops used for allocation concealment.	**High** As participants were aware of their treatment	**High** Not mentioned about blinding of assessor or researcher involved in the study	**Low** They have mentioned the reason for drop outs.	**Low** All the study outcomes were clearly reported	**High** Data of registration trial is not given.
**10. Silva F et al; 2007 Brazil**	**Low** Randomization process was mentioned i.e., **Computer-based randomization**	**Low** List no. was covered by a tab, which hide the assigned group of the next subject to researcher.	**Low** Reason mentioned that blinding of participants is not possible in the study (due to water immersion)	**High** Not mentioned about blinding of assessor or researcher involved in the study	**Low** They have mentioned the reason for drop outs.	**Low** All the study outcomes were clearly reported	**High** Data of registration trial is not given.

Supplementary file-5: Funnel plot of comparison: Heat Therapy versus RC, Outcome: Apgar score at 5th min.

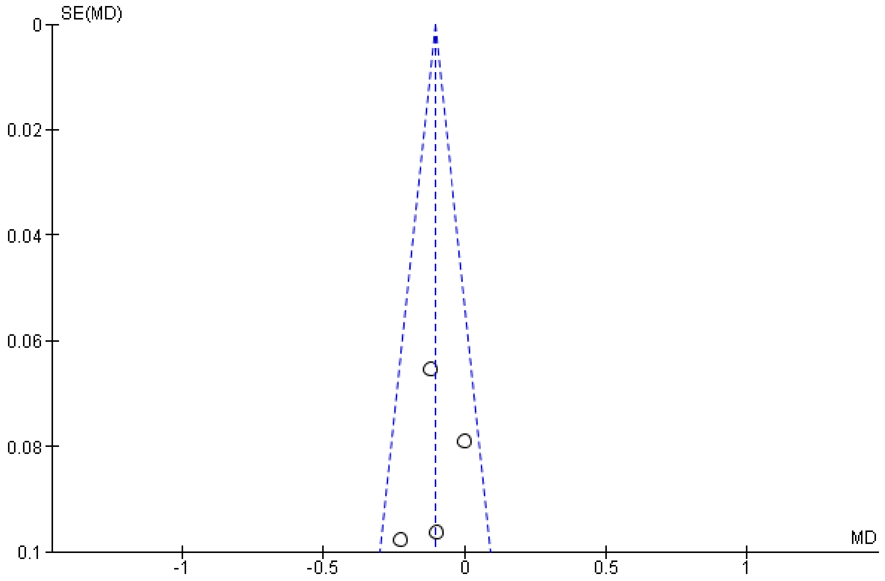

Supplementary-file 6: Forest plot of comparison: HT versus RC, Outcome: Apgar score at 1st min.

	Heat			Control				Mean Difference
Study or Subgroup	Mean	SD	Total	Mean	SD	Total	Weight	IV, Random, 95% CI
Akbarzadeh M et al; 2018, Iran	8.95	0.19	74	8.97	0.162	75	82.7%	-0.02 [-0.08, 0.04]
Eckert K et al; 2001, South Australia	9	1.7	137	9	1.5	137	1.8%	0.00 [-0.38, 0.38]
Silva F et al; 2007, Brazil	8.7	0.5	54	8.8	0.5	54	7.5%	-0.10 [-0.29, 0.09]
Tarrats L et al; 2019, Spain	8.66	1.27	67	8.98	0.48	67	2.5%	-0.32 [-0.65, 0.01]
Yazdkhasti M et al;2018, Iran	8.68	0.47	35	8.68	0.47	34	5.4%	0.00 [-0.22, 0.22]
**Total (95% CI)**			**367**			**367**	**100.0%**	**-0.03 [-0.08, 0.02]**

Heterogeneity: Tau² = 0.00; Chi² = 3.79, df = 4 (P = 0.43); I² = 0%
Test for overall effect: Z = 1.22 (P = 0.22)

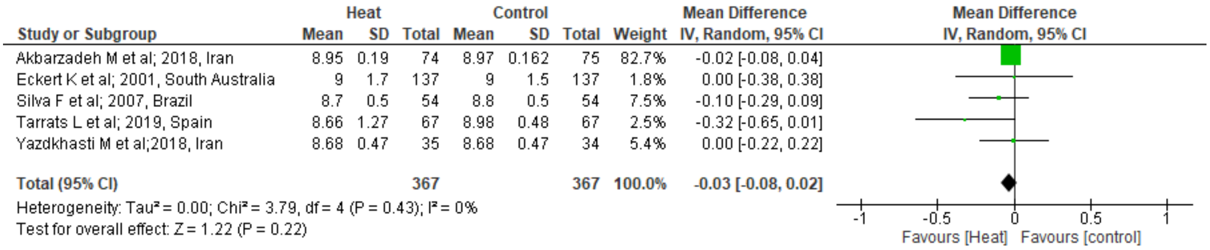

Supplementary file-7: Forest plot of comparison: HT versus RC, Outcome: Apgar score at 5th min.

Study or Subgroup	Heat Mean	Heat SD	Heat Total	Control Mean	Control SD	Control Total	Weight	Mean Difference IV, Random, 95% CI
Akbarzadeh M et al; 2018, Iran	9.9	0.11	74	9.89	0.115	75	34.7%	0.01 [-0.03, 0.05]
Eckert K et al; 2001, South Australia	9	0.7	137	9	0.6	137	17.3%	0.00 [-0.15, 0.15]
Silva F et al; 2007, Brazil	9.4	0.5	54	9.5	0.5	54	13.7%	-0.10 [-0.29, 0.09]
Tarrats L et al; 2019, Spain	9.75	0.79	67	9.98	0.12	67	13.5%	-0.23 [-0.42, -0.04]
Yazdkhasti M et al;2018, Iran	9.85	0.35	35	9.97	0.16	34	20.8%	-0.12 [-0.25, 0.01]
**Total (95% CI)**			**367**			**367**	**100.0%**	**-0.07 [-0.15, 0.02]**

Heterogeneity: Tau² = 0.01; Chi² = 9.99, df = 4 (P = 0.04); I² = 60%
Test for overall effect: Z = 1.47 (P = 0.14)

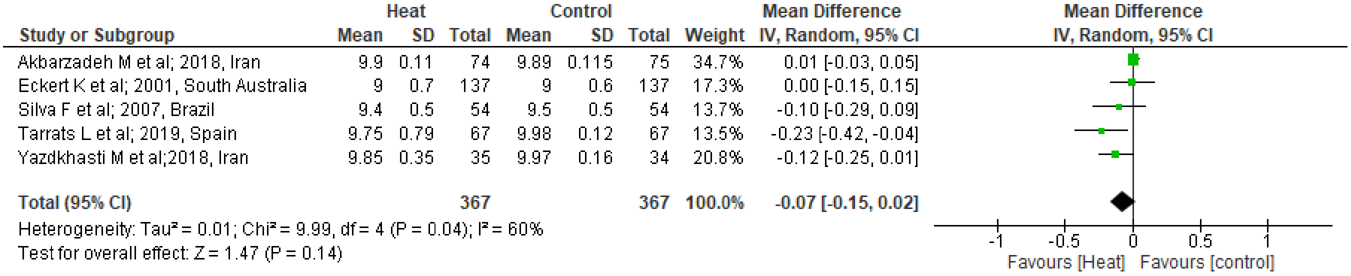

Supplementary file-8: Forest plot of comparison: HT versus RC, Outcome: Apgar score at 5th min.

Study or Subgroup	Heat therapy Mean	Heat therapy SD	Heat therapy Total	Control Mean	Control SD	Control Total	Weight	Mean Difference IV, Random, 95% CI
Eckert K et al; 2001, South Australia	9	0.7	137	9	0.6	137	26.6%	0.00 [-0.15, 0.15]
Silva F et al; 2007, Brazil	9.4	0.5	54	9.5	0.5	54	18.7%	-0.10 [-0.29, 0.09]
Tarrats L et al; 2019, Spain	9.75	0.79	67	9.98	0.12	67	18.3%	-0.23 [-0.42, -0.04]
Yazdkhasti M et al;2018, Iran	9.85	0.35	35	9.97	0.16	34	36.4%	-0.12 [-0.25, 0.01]
**Total (95% CI)**			**293**			**292**	**100.0%**	**-0.10 [-0.19, -0.02]**

Heterogeneity: Tau² = 0.00; Chi² = 3.47, df = 3 (P = 0.32); I² = 14%
Test for overall effect: Z = 2.37 (P = 0.02)

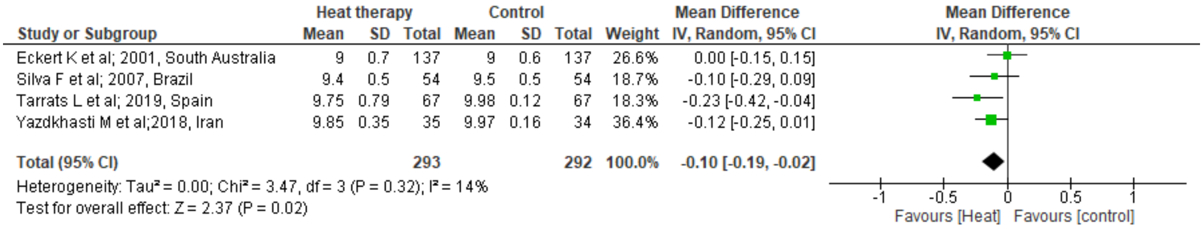

© 2022 Goswami S. et al.
